# Non-linear relationship between the first meal time of the day and gallstone incidence in American adults: a population-based cross-sectional study

**DOI:** 10.3389/fnut.2024.1521707

**Published:** 2024-12-16

**Authors:** Tiange Sun, Lidong Zhang, Ying Lu, Xianwen Zhang, Jinhao Cui, Tongheng Yang, Dan Zhang, Bowen Zheng, Shuguo Zheng

**Affiliations:** ^1^Department of General Thoracic and Urological Surgery, 78th Group Military Hospital of the PLA Army, Mudanjiang, China; ^2^Institute of Hepatobiliary Surgery, First Affiliated Hospital, Army Medical University, Chongqing, China

**Keywords:** first meal time, gallstone, American adults, NHANES, non-linear relationship, threshold effect, dietary

## Abstract

**Background:**

Irregular meal time is associated with gallstones. The time–dose effect between meal time and gallstone formation remains unknown.

**Objective:**

This study aimed to investigate the association between the first meal time (FMT) of the day and the prevalence of gallstones.

**Methods:**

Based on data from the National Health and Nutrition Examination Survey from 2017 to March 2020, the associations between the FMT of the day and the prevalence of gallstones were analyzed via multivariable logistic regression, restricted cubic spline curves, subgroup analysis, and interaction tests.

**Results:**

A total of 6,547 participants were included. The fully adjusted model indicated a positive correlation between the FMT of the day and the prevalence of gallstones (odds ratio [OR] = 1.05, 95% confidence interval [CI] = 1.02 ~ 1.08); this association was consistent across subgroups. The risk of developing gallstones was the greatest when the FMT was between 09:00 and 14:00 (OR [95% CI] = 1.49 [1.24 ~ 1.77]). There was a non-linear relationship between the FMT and gallstone incidence (*P* for non-linearity = 0.042), with an inflection point at 13.4 h. After the 13.4-h mark, the risk of developing gallstones did not increase further.

**Conclusion:**

The FMT of the day is positively correlated with the prevalence of gallstones, and there is a non-linear relationship and threshold effect between the two. Skipping breakfast is associated with a greater risk of developing gallstones. This study provides new evidence for the dietary prevention of gallstones.

## 1 Introduction

Gallstones are a common digestive system disease, and there are significant differences in prevalence across different regions and populations. The prevalence is higher in developed countries (1). In the United States, more than 20 million people have gallstones, making it the second most common gastrointestinal, liver, and pancreatic disease diagnosed, accounting for approximately 20% of all related diseases (2–4). The prevalence in Europe is slightly higher, especially in Scandinavian countries, where it can exceed 20% (2). Among American Indians, the prevalence of gallstones is as high as 70%, whereas it is 10–15% among adult Caucasians (2). In contrast, the prevalence among Asian populations is relatively low (5). Most people with gallstones are asymptomatic, but 3–8% of patients may develop complications such as cholecystitis, cholangitis, and pancreatitis, which require surgical treatment (6). In the United States, the medical costs associated with gallstones amount to billions of dollars annually, imposing a significant economic burden on public health (2).

Gallstones can be classified into cholesterol stones, pigment stones, and mixed stones. In developed countries, cholesterol stones account for approximately 80–85% of all cases (2). Risk factors for gallstones include age > 40 years, female sex, obesity, pregnancy, short-acting contraceptive use, diabetes and metabolic syndrome (7–10). Additionally, diet is an important factor influencing gallstone formation. A diet high in fat, high in cholesterol, and low in fiber increases the risk of developing gallstones (11, 12). Irregular meal times can lead to irregular gallbladder emptying, which may increase the retention time of bile in the gallbladder, which can lead to increased bile concentrations and thus an increased risk of stone formation. A study has shown that those who regularly work at night, participate in nighttime entertainment and food consumption, or work long shifts are at a greater risk of developing gallstone disease (13).

The overall association between the overnight fasting period and gallstones has been elucidated (14–16). However, the specific time–dose effect between meal time and gallstones still requires further investigation. This study aimed to explore the association between the first meal time (FMT) of the day and the prevalence of gallstones to provide new evidence for the prevention of gallstones.

## 2 Materials and methods

### 2.1 Study design and population

**Figure 1:**
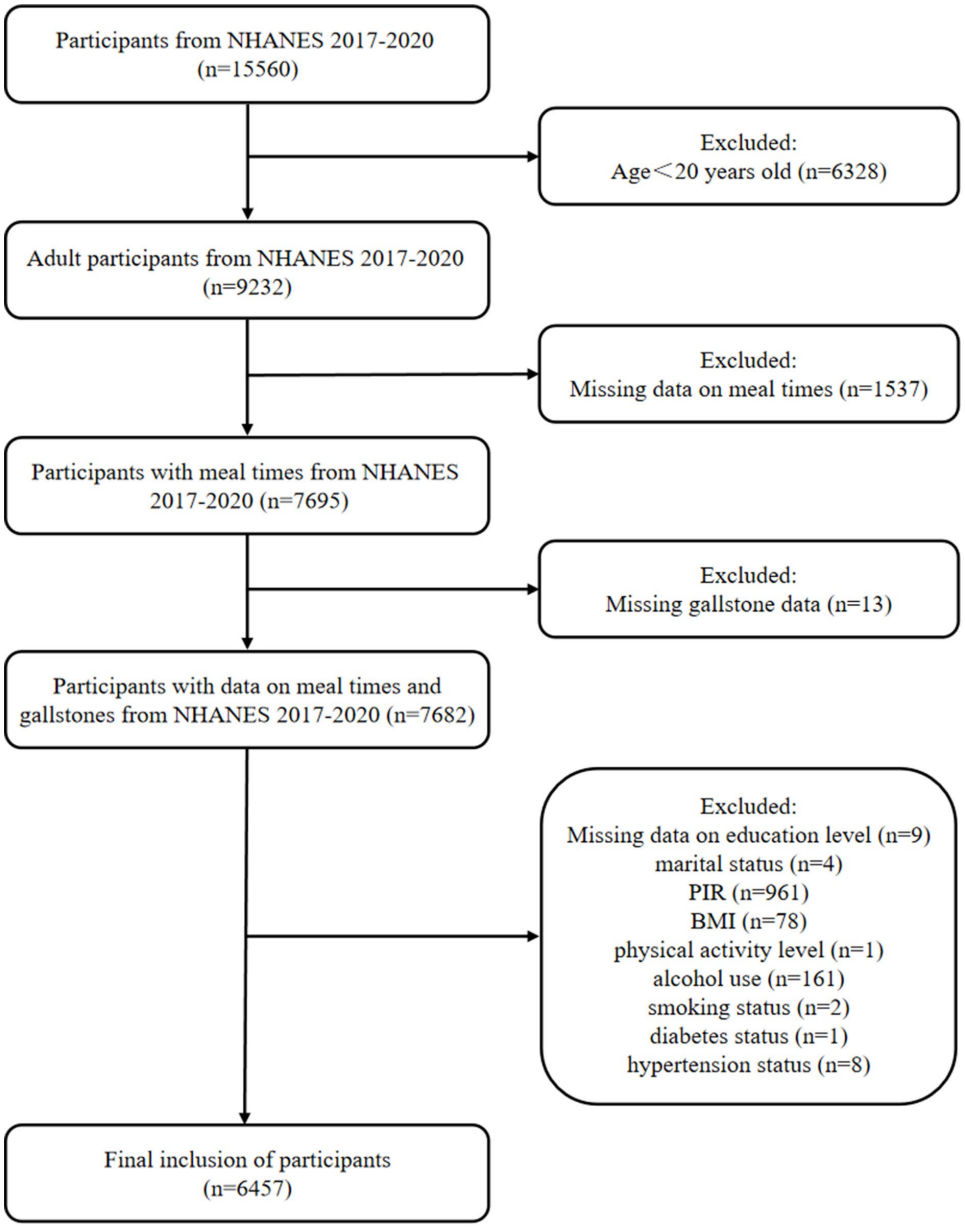
Flow chart of participant selection. PIR, ratio of family income to poverty.

The National Health and Nutrition Examination Survey (NHANES) is a stratified, multistage design, randomized sample study combining interviews, physical examinations, and laboratory tests. All research was conducted in accordance with both the Declarations of Helsinki and Istanbul. The study protocol was approved by the National Center for Health Statistics Research Ethics Review Board. Informed consent was obtained from each participant prior to data collection. This study collected data from 2017 to March 2020 and included a total of 15,560 participants, excluding 6,328 participants younger than 20 years old, 1,537 participants with missing data on meal times, 13 participants with missing gallstone data, and 1,225 participants with other missing covariates. Finally, 6,457 participants were included, of whom 695 reported the presence of gallstones (Figure 1).

### 2.2 FMT and gallstones

The exposure variable for this study was the FMT of the day, which was defined as the time of the first oral intake of solid or liquid food on that day, obtained through the first 24-h dietary review, i.e., individual foods. After determining the FMT of the day, the corresponding food code was identified and then compared with the United States Department of Agriculture food code file (Supplementary USDA Food Code) in the NHANES database to determine the food type. If the food type was water, tea, wine, coffee, juice, soda, a sports drink, or an energy drink, it was not considered a meal. Although some of these foods contain sugar, electrolytes, and/or vitamins, they lack comprehensive nutrients such as proteins, fats, and dietary fibers; they are rapidly absorbed and excreted in the digestive system, failing to provide sustained energy and satiety (17–19). Therefore, the consumption of these foods is not considered a meal. Gallstones are the outcome variable in this study and the diagnosis of gallstones is based on self-reported data from a questionnaire, which asks, “Has a doctor or other health professional ever told {you/SP} that {you/s/he} had gallstones?”.

### 2.3 Covariates

The covariates in this study refer to previous studies (6, 15, 20–22) and include sex, age, race, education level, marital status, the ratio of family income to poverty (PIR), BMI, physical activity level, alcohol use, smoking status, diabetes status, hypertension status, energy level, protein intake, carbohydrate intake, dietary fiber intake, total fat intake, total saturated fatty acid intake, and cholesterol intake. Physical activity is a binary variable, with “yes” indicating engaging in any moderate-intensity exercise, fitness, or recreational activities that cause a slight increase in breathing or heart rate within a week. Alcohol use is a binary variable, with “yes” indicating having consumed at least one drink of any kind of alcohol in one’s lifetime. Smoking status is a binary variable, with “yes” indicating having smoked at least 100 cigarettes in one’s lifetime. Diabetes is defined as a self-reported diagnosis or the current use of diabetes medication. Hypertension is defined similarly to diabetes. Energy and nutrient covariates were obtained through the first 24-h dietary review-total nutrient intake assessment.

### 2.4 Statistical analysis

Categorical variables are presented as frequencies (percentages) and were analyzed via the chi-square test. Continuous variables are reported as medians (1st quartile, 3rd quartile) and were analyzed via the Mann–Whitney *U* test. Multivariate logistic regression was used to analyze the association between the FMT and the prevalence of gallstones. Model 1 did not adjust for covariates. Model 2 was adjusted for sex, age, and race. Model 3 was adjusted for all covariates. The FMT was also converted into categorical variables, which were divided into 00:00–09:00 (breakfast period), 09:00–14:00 (lunch period), 14:00–20:00 (dinner period), and 20:00–24:00 (late-night snack period), for further analysis of its association with gallstones. Restricted cubic spline (RCS) curves were used to analyze the non-linear trends and threshold effects between the FMT and gallstones. Non-linearity was tested using the likelihood ratio test. Subgroup analysis and interaction tests were conducted to explore the robustness of the association between the FMT and gallstones in different subgroups. All tests were two-sided, with a *p* < 0.05 considered statistically significant. Statistical analyses were conducted via EmpowerStats (version 4.1) and R Software (version 4.3.0).

## 3 Results

### 3.1 Baseline characteristics

Table 1 presents the baseline characteristics of the participants. Among the 6,457 adult participants, 3,318 (51.39%) were female, 2,443 (37.83%) were Non-Hispanic White, and 695 (10.76%) had gallstones. The median age was 52.00 (36.00, 64.00) years, and the median first mealtime was 9:00 (7:50, 11:00) hours. There were statistically significant differences (*p* < 0.05) between the two groups in terms of age, BMI, FMT, energy level, protein intake, carbohydrate intake, dietary fiber intake, total fat intake, total saturated fatty acid intake, cholesterol intake, sex, race, marital status, physical activity level, smoking status, diabetes status, and hypertension status.

**Table 1:** Baseline characteristics of participants.

Variables	Total (*n* = 6,457)	Non-gallstone (*n* = 5,762)	Gallstone (*n* = 695)	*P*
Age (years)	52.00 (36.00, 64.00)	50.00 (35.00, 63.00)	60.00 (46.00, 70.00)	<0.001
Sex, *n*(%)				<0.001
Female	3,318 (51.39)	2,818 (48.91)	500 (71.94)	
Male	3,139 (48.61)	2,944 (51.09)	195 (28.06)	
Race, *n*(%)				<0.001
Mexican American	727 (11.26)	645 (11.19)	82 (11.80)	
Other Hispanic	616 (9.54)	537 (9.32)	79 (11.37)	
Non-Hispanic White	2,443 (37.83)	2,126 (36.90)	317 (45.61)	
Non-Hispanic Black	1,669 (25.85)	1,537 (26.67)	132 (18.99)	
Other Race	1,002 (15.52)	917 (15.91)	85 (12.23)	
Education level, *n*(%)				0.423
Below high school	1,053 (16.31)	936 (16.24)	117 (16.83)	
High school	1,553 (24.05)	1,374 (23.85)	179 (25.76)	
Above high school	3,851 (59.64)	3,452 (59.91)	399 (57.41)	
Marital status, *n*(%)				<0.001
Cohabitation	3,778 (58.51)	3,364 (58.38)	414 (59.57)	
Living alone	1,452 (22.49)	1,253 (21.75)	199 (28.63)	
Never married	1,227 (19.00)	1,145 (19.87)	82 (11.80)	
PIR	2.40 (1.26, 4.56)	2.41 (1.25, 4.57)	2.25 (1.33, 4.25)	0.573
BMI (Kg/m^2^)	28.90 (25.00, 34.10)	28.60 (24.70, 33.60)	32.00 (27.80, 38.05)	<0.001
Physical activity level, *n*(%)				<0.001
No	3,825 (59.24)	3,373 (58.54)	452 (65.04)	
Yes	2,632 (40.76)	2,389 (41.46)	243 (34.96)	
Alcohol use, *n*(%)				0.899
No	540 (8.36)	481 (8.35)	59 (8.49)	
Yes	5,917 (91.64)	5,281 (91.65)	636 (91.51)	
Smoking status, *n*(%)				0.001
No	3,694 (57.21)	3,336 (57.90)	358 (51.51)	
Yes	2,763 (42.79)	2,426 (42.10)	337 (48.49)	
Diabetes status, *n*(%)				<0.001
No	5,292 (81.96)	4,802 (83.34)	490 (70.50)	
Borderline	181 (2.80)	160 (2.78)	21 (3.02)	
Yes	984 (15.24)	800 (13.88)	184 (26.47)	
Hypertension status, *n*(%)				<0.001
No	3,983 (61.68)	3,664 (63.59)	319 (45.90)	
Yes	2,474 (38.32)	2098 (36.41)	376 (54.10)	
FMT (hours)	9.00 (7.50, 11.00)	9.00 (7.50, 11.00)	9.00 (8.00, 11.00)	0.003
Energy level (kcal)	1976.00 (1443.00, 2642.00)	2000.50 (1463.25, 2668.75)	1773.00 (1337.50, 2340.00)	<0.001
Protein intake (g)	72.08 (51.60, 100.48)	73.23 (52.25, 102.20)	62.93 (46.13, 89.20)	<0.001
Carbohydrate intake (g)	226.55 (161.94, 312.04)	228.24 (163.60, 314.54)	211.06 (147.59, 291.34)	<0.001
Dietary fiber intake (g)	14.20 (9.00, 21.40)	14.40 (9.10, 21.60)	12.80 (8.20, 19.00)	<0.001
Total fat intake (g)	79.69 (53.88, 113.08)	80.65 (54.34, 114.58)	72.77 (50.77, 100.79)	<0.001
Total saturated fatty acids intake (g)	24.72 (15.86, 36.85)	25.07 (15.89, 37.11)	22.11 (15.49, 33.48)	<0.001
Cholesterol intake (mg)	247.00 (135.00, 432.00)	251.00 (138.00, 435.00)	214.00 (115.00, 403.00)	<0.001

Variables are presented as frequencies (percentages) or medians (1st quartile, 3rd quartile). The baseline characteristics of participants were analyzed via the chi-square test and the Mann–Whitney *U* test. Sex and race are self-reported. FMT, first meal time; PIR, ratio of family income to poverty.

### 3.2 Association between the FMT and the prevalence of gallstones

Table 2 shows the relationship between the FMT and the presence of gallstones. The unadjusted model (OR [95% CI] = 1.03 [1.00 ~ 1.05]), the partially adjusted model (OR [95% CI] = 1.06 [1.03 ~ 1.09]), and the fully adjusted model (OR [95% CI] = 1.05 [1.02 ~ 1.08]) all indicated a positive correlation between the FMT and gallstones. The fully adjusted model shows that for every one-hour delay in the FMT, the risk of developing gallstones increases by 5%. When the FMT was converted into categorical variables for further analysis, the fully adjusted model revealed that, compared with the FMT between 00:00 and 09:00 (breakfast period), the risk of gallstones increased by 49% (OR [95% CI] = 1.49 [1.24 ~ 1.77]) for the FMT between 09:00 and 14:00 (lunch period), by 35% (OR [95% CI] = 1.35 [0.96 ~ 1.91]) for the FMT between 14:00 and 20:00 (dinner period), and by 33% (OR [95% CI] = 1.33 [0.46 ~ 3.84]) for the FMT between 20:00 and 24:00 (late-night snack period). The risk of gallstones tended to decrease with increasing FMT, and the trend test indicated that this decreasing trend was statistically significant (*P* for trend = 0.008).

**Table 2:** Association between the first meal time and the prevalence of gallstones.

Variables	Model 1^1^			Model 2^2^			Model 3^3^		
	OR (95% CI)	*P*	*P* for trend	OR (95% CI)	*P*	*P* for trend	OR (95% CI)	*P*	*P* for trend
FMT (continuous)	1.03 (1.00 ~ 1.05)	0.033		1.06 (1.03 ~ 1.09)	<0.001		1.05 (1.02 ~ 1.08)	0.001	
FMT (categorical)									
00:00–09:00	1.00 (Reference)			1.00 (Reference)		<0.001	1.00 (Reference)		
09:00–14:00	1.33 (1.13 ~ 1.57)	<0.001	0.382	1.55 (1.31 ~ 1.85)	<0.001		1.49 (1.20 ~ 1.77)	<0.001	0.008
14:00–20:00	1.06 (0.78 ~ 1.45)	0.701		1.54 (1.11 ~ 2.14)	0.009		1.35 (0.96 ~ 1.91)	0.082	
20:00–24:00	0.75 (0.27 ~ 2.08)	0.577		1.43 (0.50 ~ 4.06)	0.507		1.33 (0.46 ~ 3.84)	0.597	

Multivariate logistic regression and trend test were used to analyze the association between the FMT and the prevalence of gallstones. ^1^No covariates were adjusted. ^2^Sex, age, race were adjusted. ^3^Sex, age, race, education level, marital status, PIR, BMI, physical activity level, alcohol use, smoking status, diabetes status, hypertension status, energy level, protein intake, carbohydrate intake, dietary fiber intake, total fat intake, total saturated fatty acid intake, and cholesterol intake were adjusted. FMT, first meal time; PIR, ratio of family income to poverty.

### 3.3 Non-linear trend and threshold effect analysis

**Figure 2:**
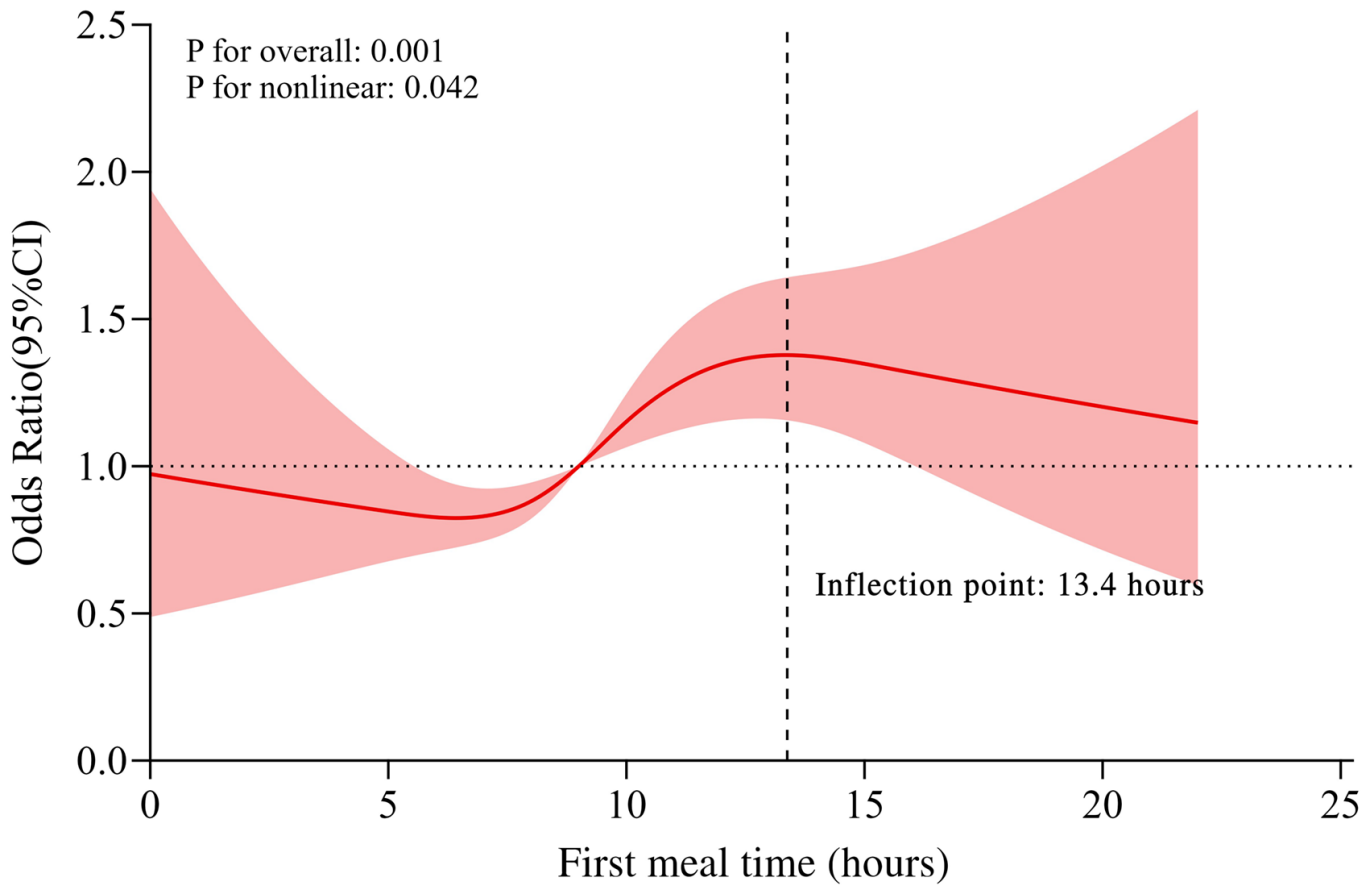
The time–dose effect between the first meal and the prevalence of gallstones. The RCS curve based on the fully adjusted model was plotted to further explore the non-linear trend and threshold effect between the FMT and the presence of gallstones. Sex, age, race, education level, marital status, PIR, BMI, physical activity level, alcohol use, smoking status, diabetes status, hypertension status, energy level, protein intake, carbohydrate intake, dietary fiber intake, total fat intake, total saturated fatty acid intake, and cholesterol intake were adjusted. The red solid line represents the estimated values, and the shaded area represents the 95% CI. The intersection of the vertical black dashed line and the red solid line represents the inflection point. FMT, first meal time; PIR, ratio of family income to poverty; RCS, restricted cubic spline.

An RCS curve based on the fully adjusted model was plotted to further explore the non-linear trend and threshold effect between the FMT and the presence of gallstones. The results revealed a non-linear relationship between the FMT and the prevalence of gallstones (*P* for non-linearity = 0.042) (Figure 2). A two-piecewise logistic regression analysis identified an inflection point at 13.4 h. When the FMT duration was less than 13.4 h, each additional hour was associated with a 7% increase in the risk of gallstones (OR [95% CI] = 1.07 [1.03 ~ 1.12]). When the FMT duration was 13.4 h or more, the positive correlation between the FMT and gallstones was no longer significant (OR [95% CI] = 1.04 [0.90 ~ 1.19]) (Table 3).

**Table 3:** Threshold effect analysis between the first meal time and the prevalence of gallstones.

Two-piecewise logistic regression model^1^	OR (95% CI)	*P*	*P* for non-linearity
FMT ≤ 13.4 h	1.07 (1.03 ~ 1.12)	0.002	0.042
FMT > 13.4 h	1.04 (0.90 ~ 1.19)	0.617	

The two-piecewise logistic regression analysis identified threshold effect between the FMT and the presence of gallstones. ^1^Sex, age, race, education level, marital status, PIR, BMI, physical activity level, alcohol use, smoking status, diabetes status, hypertension status, energy level, protein intake, carbohydrate intake, dietary fiber intake, total fat intake, total saturated fatty acid intake, and cholesterol intake were adjusted. FMT, first meal time; PIR, ratio of family income to poverty.

### 3.4 Subgroup analysis

**Figure 3:**
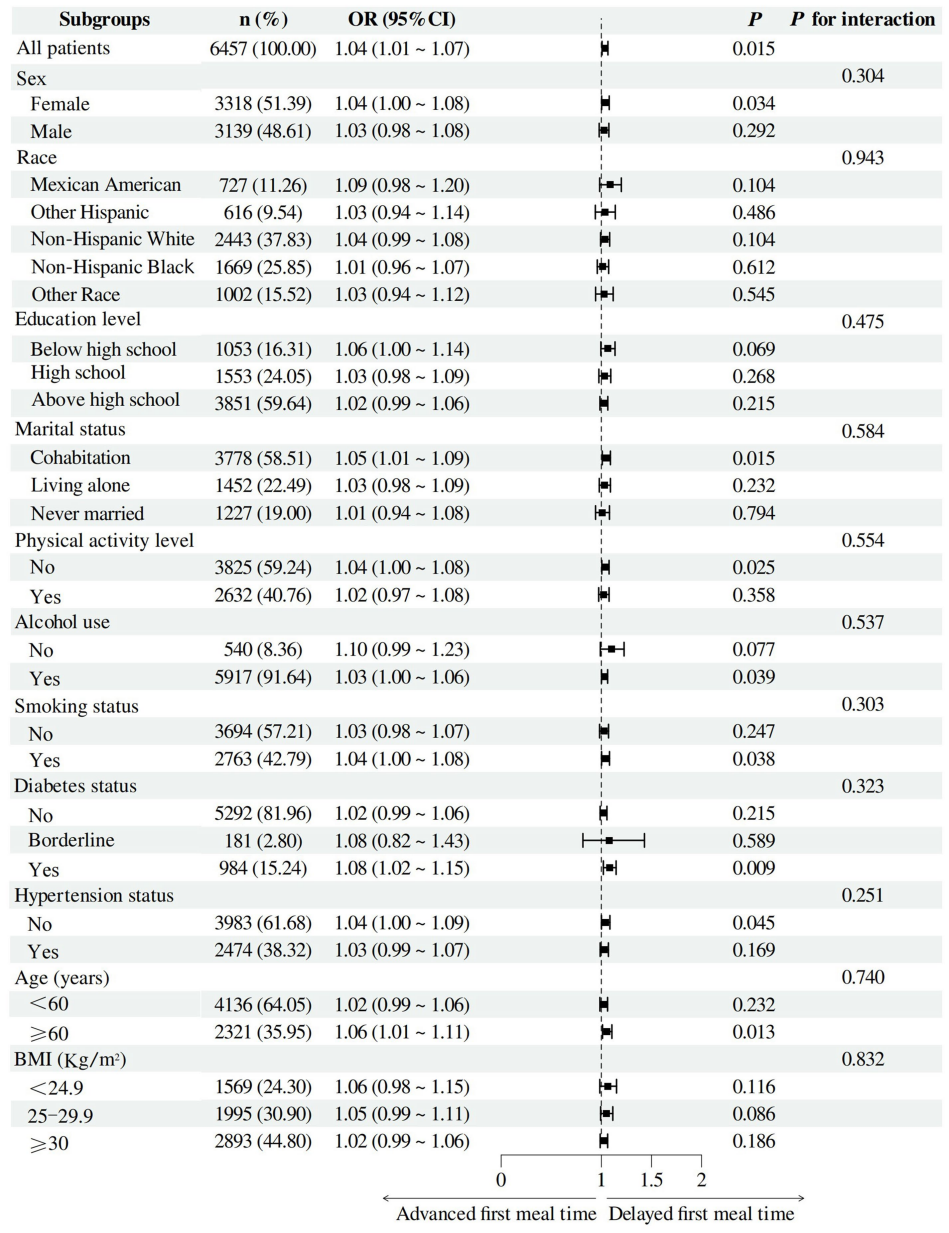
Subgroup analysis of the association between the first meal time and the prevalence of gallstones. Subgroup analysis and interaction tests were conducted to explore the robustness of the association between the FMT and gallstones in different subgroups. Sex, age, race, education level, marital status, PIR, BMI, physical activity level, alcohol use, smoking status, diabetes status, hypertension status, energy level, protein intake, carbohydrate intake, dietary fiber intake, total fat intake, total saturated fatty acid intake, and cholesterol intake were adjusted. FMT, first meal time; PIR, ratio of family income to poverty.

The participants were stratified according to sex, race, age, BMI, education level, marital status, physical activity level, alcohol use, smoking status, diabetes status, and hypertension status for subgroup analysis and interaction tests. The results revealed a significant positive correlation between the FMT and gallstones among women, married individuals, those with no exercise habits, alcohol drinkers, smokers, individuals with diabetes, those without hypertension, and those aged 60 years or older. However, the interactions between the FMT and these subgroups were not significant. Overall, the positive correlation between the FMT and gallstones was consistent across different subgroups (Figure 3).

## 4 Discussion

This study examined the time–dose effect between the FMT of the day and the presence of gallstones in a large population sample. The study revealed that the FMT was positively correlated with the prevalence of gallstones and remained consistent across various subgroups. Compared with that when the FMT was between 0:00 and 09:00, the risk of gallstones was greater when the FMT was between 09:00 and 14:00, indicating that skipping breakfast was more correlated with the development of gallstones. Additionally, we found a non-linear relationship between the FMT and gallstones. When the FMT exceeded 13.4 h, the risk of developing gallstones no longer increased with further delays in the FMT.

Previous studies have shown that a prolonged overnight fasting period may increase the risk of developing gallstones. Capron et al. (14) reported in a short report that short-term prolonged fasting in French women aged 20–35 years may increase the risk of gallstone formation. Sichieri et al. (15) reported in a prospective study that the risk of hospitalization due to gallstones in American women increased with prolonged overnight fasting, with the highest risk observed for fasting periods of 14 h or more. In a cross-sectional study of an Italian population, Attili et al. (16) reported that the prevalence of gallstones was greater in individuals who fasted for more than 12 h at night than in those who fasted for less than 12 h. The results of this study revealed that the prevalence of gallstones gradually increased with the delay in the first meal of the day, which is consistent with the findings of the aforementioned studies. Prolonged fasting can cause bile to remain in the gallbladder for an extended period, during which its water content is gradually absorbed, leading to an increased bile concentration. The increased saturation of cholesterol makes it more likely to aggregate and crystallize within the gallbladder, forming stones (23, 24). Williams et al. (25) reported that among women without gallstones, 4.5% had cholesterol-saturated bile after 9 h of fasting, and this percentage increased to 54.5% after 16 h of fasting. Additionally, during fasting, bile acids are partially stored in the unemptied gallbladder, temporarily interrupting the enterohepatic circulation of bile acids. This leads to a decreased secretion rate of bile acids and an increased proportion of cholesterol in the bile, thereby increasing the risk of cholesterol precipitation and gallstone formation (26).

Further examination of the time–dose effect between the FMT and the prevalence of gallstones revealed a non-linear relationship. Compared with the FMT from 0:00–09:00, the risk of gallstones was relatively greater from 09:00–14:00, and the risk of gallstones from 14:00–20:00 and 20:00–24:00 showed a downward trend. A similar non-linear relationship was reported by Bloch et al. (26), with results that showed the average cholesterol saturation index in 9 healthy women was significantly greater after 15 h of fasting than after 10 h of fasting, with a significant decrease after 20 h of fasting. A possible explanation is that reduced hepatic cholesterol synthesis leads to unsaturated hepatic bile. In the livers of rats, the activity of the rate-limiting enzyme (hydroxymethyl-glutaryl coenzyme-A reductase) in cholesterol synthesis begins to decrease within 6–8 h of fasting, reaching its lowest level 14 h after the last meal (27–29). Gälman et al. (30) reported a possible peak in bile acid synthesis at 13:00, which alters the ratio of bile acid to cholesterol in bile and increases cholesterol solubility, possibly explaining why the risk of gallstones did not increase further after the FMT reached the inflection point of 13.4 h.

In Table 1, we observed that the median BMI of the gallstone group was higher than that of the non-gallstone group. However, participants in the gallstone group reported lower total energy, saturated fatty acids, and cholesterol intake compared to the non-gallstone group, which contrasts with findings from prior studies. One possible explanation is that participants diagnosed with gallstones may have altered their diets following medical advice, reducing their intake of calories, saturated fatty acids, and cholesterol to manage symptoms and slow the progression of gallstone. Moreover, obese participants in the gallstone group may prioritize dietary management more actively. They could adopt low-calorie, low-fat diets as a strategy to manage their weight or improve their overall health. Furthermore, when reporting their dietary intake, they might underreport their actual caloric and fat consumption, whether consciously or unconsciously. Inconsistencies in findings regarding the relationship between dietary factors and gallstone risk have also been reported in previous studies. For example, both the studies by Attili et al. (16) and Smith and Gee (31) found a negative correlation between low caloric intake and the risk of developing gallstones. Festi et al. (32) noted that very low-calorie diets in obese individuals might increase the risk of gallstone formation. Similarly, studies investigating the relationship between total fat intake and cholesterol gallstones have yielded mixed results, ranging from positive to non-significant associations (24). These discrepancies likely stem from the complexity of human dietary patterns, as well as variations in sample characteristics and overall health status.

This study has several strengths. The first is the use of a representative sample of the U.S. population from the NHANES database, with participants strictly adhering to the study protocol and being supervised by comprehensive quality control and assurance measures, thereby ensuring the reliability and accuracy of the study results. The second strength is the further exploration of the non-linear relationship and threshold effect between the first meal time of the day and the prevalence of gallbladder stones. The study’s limitations include the cross-sectional study design, which cannot establish a causal relationship between the first meal time and the prevalence of gallbladder stones. Additionally, too many variables related to gallbladder stones are included in the model to control for confounding bias. Finally, due to the inability to differentiate between gallstone compositions, our study may not fully capture the association between the FMT of the day and different types of gallstones. This limitation could restrict a complete understanding of the pathogenesis of gallstones, particularly in relation to dietary habits and lifestyle factors. We recommend that future research utilize datasets containing more comprehensive information on gallstone composition to enable a deeper investigation of these associations.

This study revealed that the FMT of the day is positively correlated with the prevalence of gallstones among U.S. adults and remains consistent across various subgroups. The risk of developing gallstones is relatively high when the FMT is between 09:00 and 14:00. There was a non-linear relationship between the FMT and gallstones, with an inflection point at 13.4 h. This research supplements previous findings, but large-scale prospective cohort studies are still needed to further validate these results.

## Data Availability

Publicly available datasets were analyzed in this study. This data can be found: https://www.cdc.gov/nchs/nhanes/index.htm.
